# The Role of Open Science Practices in Scaling Evidence-Based Prevention Programs

**DOI:** 10.1007/s11121-021-01322-8

**Published:** 2021-11-15

**Authors:** Lauren H. Supplee, Robert T. Ammerman, Anne K. Duggan, John A. List, Dana Suskind

**Affiliations:** 1 William T. Grant Foundation, New York, NY USA; 2 Cincinnati Children’s Hospital Medical Center, University of Cincinnati College of Medicine, Cincinnati, USA; 3 Johns Hopkins University, Baltimore, USA; 4 University of Chicago, Chicago, USA

**Keywords:** Evidence-based programs, Open science, Home visiting, Scaling

## Abstract

The goal of creating evidence-based programs is to scale them at sufficient breadth to support population-level improvements in critical outcomes. However, this promise is challenging to fulfill. One of the biggest issues for the field is the reduction in effect sizes seen when a program is taken to scale. This paper discusses an economic perspective that identifies the underlying incentives in the research process that lead to scale up problems and to deliver potential solutions to strengthen outcomes at scale. The principles of open science are well aligned with this goal. One prevention program that has begun to scale across the USA is early childhood home visiting. While there is substantial impact research on home visiting, overall average effect size is .10 and a recent national randomized trial found attenuated effect sizes in programs implemented under real-world conditions. The paper concludes with a case study of the relevance of the economic model and open science in developing and scaling evidence-based home visiting. The case study considers how the traditional approach for testing interventions has influenced home visiting’s evolution to date and how open science practices could have supported efforts to maintain impacts while scaling home visiting. It concludes by considering how open science can accelerate the refinement and scaling of home visiting interventions going forward, through accelerated translation of research into policy and practice.

The hope is evidence-based prevention programs, if scaled up, will shift population-level outcomes in communities. However, the challenges in scaling evidence-based programs and maintaining the promised impacts are large, both in the complex infrastructure necessary to support programs effectively (Braithwaite et al., 2018; Bold et al., 2018; Fagan et al., 2019; Greenhalgh & Paptousi, 2019) and ensuring the programs scaled can deliver those impacts (Al-Ubayldi et al., 2020a; Bangser, 2014). The problems around scale-up can lead to a waste of resources, a missed opportunity to improve people’s lives, and a diminution in the public’s trust in the scientific method’s ability to contribute to policymaking (Al-Ubayldi et al., 2020a). The science of studying scaling is critical to ensure the promise of evidence-based policy to deliver community level outcomes is fulfilled.

Implementation scientists have been exploring what features are necessary for programs to deliver at scale, and much progress has been made (e.g., Bauer et al., 2015; Bold et al., 2018; Durlak, 2015). Much of the work in implementation science focuses after a program has demonstrated efficacy in rigorous research, and the goal now is to examine adoption, implementation, and scale up (Baker, 2010; Dearing & Cox, 2018). A key feature of implementation science revolves around concerns about the “voltage effect”—treatment effect sizes observed in the original impact studies diminish substantially when the program is rolled out at larger scale (Gottfredson et al., 2015; Kilbourne et al., 2007; Weiss et al., 2014). The literature has used this cautionary tale to stress that scaling up is an intricate, complex process (Braithwaite et al., 2018), and it oftentimes implies that the optimism advertised in the original research may be unjustified (Milat et al., 2016). However, much of the literature considers the problem after the research on evidence-based programs is completed and not at the scientific system that is incentivizing the creation of evidence for scaling (Milat et al., 2013).

The paper will present an economic perspective on the incentives and disincentives in the current scientific enterprise to generate evidence that supports effective scaling of evidence-based programs. The paper briefly discusses these threats to effective scaling in the current system and provides recommendations on changes to the system, including how the use of open science practices can begin to address the identified problems. Finally, the paper concludes with a case study of early childhood home visiting that highlights the problems in the current science of scaling and how open science practices could address some of the issues.

## Incentives and Disincentives in the Scientific System

To date, economics has largely been on the sidelines of the implementation science and scaling literature, rendering itself mute on how it can enhance our understanding of the science of using science.^1^ Recently, a series of papers by Al-Ubaydli et al. (2017, 2020a, b) apply the lens of economics to the challenges of scaling evidence-based programs. Using an economic lens is useful because it pinpoints the underlying incentives inherent in the scientific system that may be leading to voltage effect drops at scale, and points to areas where improvements can be made.

Consider false positives, a key target of the open science movement, and central to decisions on whether to scale a particular program. The standard discussion around false positives is that the analyst sets the statistical error (alpha) and that determines the false positive rate (usually 5%). Examining false positives from an economic perspective reveals that this rate is much too low in practice, particularly when the goal is scaling programs. For example, while typically researchers assume the estimation error in their statistical equation is zero, economic intuition warns that it might not be true for the specific evidence-based program chosen to scale. To understand the issue, consider the phenomenon of the winner’s curse in auction theory. For concreteness, assume bidders are bidding in a government auction for the rights to drill for oil. Bidders first estimate how much oil is on the plot, how much it will cost to bring the oil to market, and they forecast a market price of oil. Bidders then place their bids, largely based on these estimates. Inevitably, some will guess too low and some too high. Yet, since the highest bidder wins the auction and has to pay their bid, the “winner” is guaranteed to lose money because they overestimated the value of drilling on the plot.

Policymakers choosing which “winning policies” to scale hold similar underlying mechanics because researchers who deliver the largest effect sizes are most likely to be noticed by policymakers. Indeed, to make matters worse, we are usually comforted when there are more researchers working on a problem because we believe we are more likely to find out the truth, but in this case, as the number of scientists studying related interventions increases, the “winning program” (i.e., the program chosen by policymakers) will be overvalued even more because there will be more chances for an extreme draw. That is, the nature of the winner’s curse increases with the number of researchers (at least in the short-run), leading to an inferential error. Thus, the false positive rate will be larger than we believe when initially setting alpha to 0.05 (see Al-Ubaydli et al., 2020a).

A second channel of bias that leads to a higher false positive rate is the choice of sample population. Scientists desire to report both replicable findings and important treatment effects. When they place non-zero weight on each, it is important to recognize that the researcher could choose their subject pool with knowledge regarding the unique attributes of the participants compared to other parties who were not involved in the experiment. For example, they can strategically choose a sample population that yields a large treatment effect. In addition, if participants with the largest expected benefits from the program are more likely to sign up, participate, and comply, a scientist who maximizes their sample size to a fixed budget constraint of their grant may be inadvertently maximizing the treatment effect size, and subsequently presenting results that may not scale. Both selection effects (one nefarious, the other perhaps not) lead to higher false positive rates (similar insights emerge if one considers representativeness of the contexts in which studies are tested such as schools or agencies). While this continues to be a problem in the literature at large, there is a growing movement in the field towards more pragmatic trials and frameworks for transparently reporting the representativeness of study populations that may begin to address this problem (Curran et al., 2012; Loudon et al., 2015).

Finally, because of publication bias (the practice in which journals overwhelmingly publish studies that have large, surprising results with low p-values), and preference of funders and government officials in the constant search for interventions with significant treatment effects, there is an incentive not to apply all appropriate data analysis techniques. Strategies such as failing to correct for multiple testing (List et al., 2019) and re-analyzing data in multiple ways to generate a desired result (also known as p-hacking), and the like all lead to a higher false positive rate than the commonly advertised 5% rate.

## Economic Model on Scaling

Scaling up promising programs into effective policies is a complex, dynamic process that begins with the market for scientific knowledge (Al-Ubaydli et al., 2020b).^2^ The knowledge market has three major players: policymakers, researchers, and community members. Policymakers fund the initial research and implement policies to provide the greatest benefit to the population within time, money, and resource constraints. Researchers conduct experiments to evaluate programs and publish the findings in academic journals. Community members receive rewards for participating in research studies and benefit from the programs the government implements. The needs of all three stakeholders — researchers, community members, and policymakers — affect the available economic model on scaling. This article will focus most closely on researchers and policymakers.

Looking at challenges related to scaling using an economic perspective, there are five aspects to an evidence-based program that policymakers want to understand before scaling a program: (1) when does evidence become actionable (appropriate statistical inference), (2) properties of the population (how representative are the participants in the research to the ultimate beneficiaries), (3) properties of the situation (how representative are the situations to the ultimate implementation contexts), (4) spillover (how much do effects of the program spillover into non-participating populations or contexts), and (5) marginal cost considerations (how much do costs change as we scale a program). Al-Ubaydli et al. (2020a) argue that until these five areas and their underpinnings are fully understood and recognized by researchers and policymakers, the threats to scalability will render achieving population-level effects through scaling evidence-based programs particularly vulnerable (List, 2022, denotes these as the “Five Vital Signs”).

Studies generated through the research enterprise contribute to the five elements described above. Understanding the sources of these elements and the incentives and disincentives in the system related to these elements is necessary to identify potential solutions to attenuating voltage effects. None of these elements are new on their own, yet the field rarely discusses their underpinnings, how the research process itself incentivizes threats to scalability, and how open science practices can support addressing the threats to scalability.

## Open Science Solutions to the Issues in the Economic Model on Scaling

Like the incentives and disincentives an economic perspective on scaling aims to address, open science acknowledges the potential for bias in research and aims to incentivize high quality science through environmental mechanisms (Wagenmakers et al., 2012). Funding and policy are necessary conditions for scaling evidence-based programs (Fagan et al., 2019); thus, policymakers must have trust in science. Open science supports decision-makers in assessing the credibility and rigor of the work (Standen, 2019). At the same time, the way studies are designed can offer opportunities to overcome threats to scaling. Open science acknowledges that study quality and reporting are within researchers’ control, but the results obtained are not (Frankenhaus & Nettle, 2018). While open science practices are well aligned with all five threats within the economic model on scaling, our discussion will focus on the connection between open science practices, specifically replication and pre-registration to support incentives and disincentives related to the first three threats in the economic model on scaling: actionable evidence, properties of the population, and properties of the situation.

### Replication

One way to increase confidence in the findings is replication of research. Open science values not only the replication itself but the conditions that support quality replication (e.g. transparency in methods through preregistration or open methods). In line with open science, the economic model on scaling calls for replication of findings to ensure the programs chosen to scale maintain their desired impacts. That is, the model highlights the power of *coordinated replications* to enhance knowledge creation and production of scalable insights, which is distinct from many researchers working on a problem without such coordination. Another open science strategy that addresses threats to scalability is the use of open data and code to encourage external researchers to replicate the findings. Open data and code shine a light on problems with analysis and reporting of findings, including genuine mistakes (Wicherts et al., 2011). Unfortunately, the current knowledge creation marketplace does not incentivize replication.

First, changes are needed within the knowledge creation market itself. For example, to shift the incentives in the system to producing more replicable work, we must recognize that the incentives within the knowledge creation system are designed such that once a study has been published, the original investigators and others have little incentive to replicate the findings. The reason is that the returns from replicating published work are generally low. This is problematic, as new and surprising findings may be false positives simply due to the mechanics of statistical inference outlined above (Dreber et al., 2015; Maniadis et al., 2017).

Currently, the research system discourages replications (Al-Ubaydli et al., 2020b). Koole and Lakens (2012) note the incentive structure in psychology is toward individual scientists with their own body of research and not as much on the scientific field in general to build and replicate knowledge. A recommendation in open science for changing the incentives towards replication is co-citing (when two documents cite each other) or co-publishing (sharing publications) to increase the stature of the findings and shift the norm from individual to group responsibility (Butera et al., 2020; Koole & Lakens, 2012). Another approach is to design simple replication mechanisms that generate mutually beneficial gains from trade among the authors of a novel study. Butera et al. (2020) propose one such approach, whereby upon completing an initial study, the original researchers write a working paper version of their research. While they can share their working paper online, they commit to never submitting the work to a journal for publication. They instead invite other researchers to coauthor and publish a second, yet-to-be-written paper, provided researchers are willing to replicate independently the experimental protocol. Once the team is established, but before the replications begin, the replication protocol is preregistered and referenced back in the first working paper. This guarantees that all replications, whether successful or unsuccessful, are properly recognized. The team of researchers then writes the second paper, which includes all replications, and submits to an academic journal.

Another strategy is to leverage multiple trials to learn about the variation of program impacts across both populational and situational dimensions. In other words, before recommending scaling a particular program, researchers should understand the program effects across subsets of the population and characteristics of the situation to understand who should receive the program and where/how it should be implemented (Orr et al., 2019; Stuart et al., 2015). An example of this strategy is the USA’s National Institutes of Health Early Intervention to Promote Cardiovascular Health of Mothers and Children initiative including low and high resourced, geographically diverse clinical, or community-sites within a multi-center or cluster randomized trials for cardio-vascular health interventions (RFA-HL-22-007: Early Intervention to Promote Cardiovascular Health of Mothers and Children (ENRICH) Multisite Clinical Centers (Collaborative UG3/UH3 Clinical Trial Required) (https://grants.nih.gov/grants/guide/rfa-files/RFA-HL-22-007.html)).

Finally, a way to revise the incentive structure in the knowledge creation system is through increasing the value of replications for tenure and promotion (Al-Ubaydli et al., 2020b). While the open science practice of replication is laudable, it needs to include the other incentives and disincentives in the scientific system. This means that we need to establish adequate rewards to scholars for designing research that can be independently replicated—tying tenure decisions, research funding, and the like to such research (i.e., increasing the demand for replicable work). Likewise, to increase the supply of replications, we should reward scholars’ replications in tenure and promotion decisions and providing research funding specifically for replication work.

### Pre-registration

Pre-registration is another way to influence the knowledge production system. Pre-registration is the practice of publicly stating the research questions and study design, and sometimes analysis plans, prior to conducting the study. Pre-registration aims to increase credibility of the research and analysis overall through transparency (Yamada, 2018).

Pre-registration is thought to reduce publication bias toward significant findings, meaning studies with null effects are more difficult to identify, which is central to the first threat to scaling, inference (Nosek & Lindsey, 2018). Pre-registration compels scientists to document confirmatory and exploratory findings (Wagenmaker et al., 2012); through this, research consumers are clear which tests were planned and which were exploratory. Al-Ubaydli et al. (2020b) assert that in the economic model on scaling the publication of all findings, including null findings, is critical to inform policy decisions.

The knowledge creation system would need to change the incentive structure to support this goal. One strategy is registered reports in which the study publishes the design for peer review prior to study initiation (Chambers, 2019), making it possible to ascertain whether study execution deviated from the study plan in ways that might meaningfully affect results. Publication of study protocols provides incentives for researchers by increasing their number of publications and by increasing the likelihood results will be published, regardless of the nature of those results. Study protocols eliminate the “file drawer” problem (not all results are published) and p-hacking in that studies are accepted prior to results being available, meaning researchers do not need to worry about not being able to publish if the trial reveals null findings.

Another way in which study protocols can also support the goals of the economic model on scaling is by improving the representativeness of the populations and situations included in trials, such as factors highlighted in the Pragmatic-Explanatory Continuum Indicator Summary tool (PRECIS-2 - Home Page (https://www.precis-2.org/) and the increase in the use of hybrid effectiveness trials (Curran et al., 2012). If more researchers incorporate scientific practices to promote scaling, including taking into account the properties of the populations and situations (two threats to scaling), the peer review process in study protocols can push scientists to design more representative trials. Having pre-registration information allows other researchers to understand how representative (or not) the populations and situations are in the executed study. For example, following List (2020), in terms of sample selection, the author should report clearly how selection of subjects occurred in two stages. First, provide details on the representativeness of the studied group compared to the target population. Second, provide details of whether the study group is representative of the target population in terms of relevant observables that might impact preferences, beliefs, or individual constraints and how that might impact generalizability. Alongside that information, the researcher would report in their final analysis attrition and participant and implementer compliance rates. This includes documenting reasons for attrition and non-compliance such as motivational or incentive differences between groups. This will provide a sense of whether the subject pool is representative and whether the program will successfully scale.

In sum, the economic model on scaling provides insights on knowledge generation and use, including pinpointing the major threats to scaling. In doing so, the incentives and disincentives embedded in the current research system that promote and discourage creation of scalable insights are revealed. This highlights the richness of the results delivered from the economic model on scaling in that entire research agendas are rooted within each of the five vital signs the model delivers.

## Home Visiting as a Case Study of How Open Science Can Strengthen Scaling Evidence

We turn now to the relevance of the threats to the economic model on scaling and open science in developing and scaling evidence-based home visiting. This section describes how the traditional approach for testing interventions has influenced the evolution of home visiting to date, then considers how the economic model on scaling and open science can accelerate the refinement and scaling of home visiting interventions going forward.

### How the Traditional Research Approach Has Influenced Home Visiting’s Evolution to Date

Early childhood home visiting is a preventive intervention for expectant families and families with children birth to 5 years. It aims to achieve equity in health and socio-economic outcomes through education and family support during visits and by linking families with needed community resources. Investment in the USA in scaling evidence-based home visiting began in earnest in 2010 through the Maternal, Infant and Early Childhood Home Visiting Program (MIECHV) (Federal Register, 2010; Bipartisan Budget Act of 2018). MIECHV legislation calls for the majority of funding to scale up evidence-based home visiting models. MIECHV and other funding streams now support thousands of local home visiting programs across the country.

The first three decades of home visiting impact research followed a traditional course. Experts in parenting, health, early childhood development, and child welfare began to develop home visiting models in the 1970s (Weiss, 1993) when the benefits of open science practices and the threats to scaling in the economic model of scaling were neither delineated nor appreciated. Often, developers relied on non-experimental program evaluation designs. Study results were often unpublished or presented outside of standard peer-reviewed venues. Findings were mixed and sometimes contradictory, generalizability was limited, identification of common intervention elements in different models was given a low priority, mechanisms of change went largely unexamined, and replication and reproducibility were severely constrained (Sama-Miller et al., 2018). Still, early evaluation results appealed to policymakers eager for programs to promote child health and prevent child maltreatment. This demand created a strong and growing incentive to scale home visiting models early on (US Advisory Board on Child Abuse and Neglect, 1991; US General Accounting Office, 1990).

In response, several models launched dissemination arms in the 1990s. In some cases, dissemination began after experimental impact studies by the model developer. In other cases, dissemination began while the evidence from experimental studies was still being developed. In 2010, the USA’s Administration for Children and Families launched the Home Visiting Evidence of Effectiveness review (HomVEE), a systematic review of rigorous peer-reviewed and grey literature on home visiting models (Sama-Miller et al., 2018). Its purpose is to identify models with sufficient evidence of impact to be designated as “evidence-based.”

While HomVEE valued scientific rigor, it was constrained by limited information on interventions and study methods in the literature. These limitations arose because journal requirements were less stringent than those recommended by open science. Practices like pre-registration were either not available or rarely used and so key information such as identification of confirmatory outcomes was unavailable. Thus, important aspects of program design, program implementation, and study methods were not reported which constrained drawing inferences from results. Still, HomVEE had to use the available research to identify evidence-based models. In 2010, it designated seven models as evidence-based. By 2018, it had identified 28,927 studies, including 363 randomized trials of 46 home visiting models (Sama-Miller et al., 2018). Today, 19 models are designated as evidence-based, have dissemination arms, and are eligible for MIECHV Program funding.

Early impact research tested average effects of full models across highly diverse families in varied communities (Sama-Miller et al., 2018). Reports tended to focus on outcomes measured after the defined duration of program enrollment. Thus, research provided little insight on the population and situational aspects of the economic model on scaling. This constrained what could be inferred from results and the implications for scaling. Most early research failed to specify core components (the specific interventions comprising models), the underlying theories of behavior change, and mechanisms of action whereby intervention modifies behavior and, through this, achieves intended outcomes (Supplee & Duggan, 2019). A priori hypotheses were not often specified, and failure to control for multiple comparisons increased risk of false positives. There was usually little or no information to judge whether null results might be attributed to faulty implementation rather than shortcomings of the model itself (Paulsell et al., 2014). It was assumed, but not tested, that intensive, long-term intervention was critical. Studies that did report “dosage” found that many families disenrolled far sooner than intended by model developers. There was no research to identify which interventions within a model were essential nor whether what was essential varied by population or situational context.

How did states decide which models to invest in and how did HomVEE’s results inform their decisions? The usefulness of HomVEE’s results was constrained by limitations of the body of published research on which it relied. The body of research showed few positive impacts, a plethora of null results, a smattering of adverse effects, and highly mixed results across multiple studies of the same model. Not all models were independently tested. The preponderance of null or negative results was more pronounced for studies conducted by independent investigators. There was some replication of studies, but results were often divergent and hard to interpret due to different measurement, populations, and contexts. Furthermore, the studies provided evidence only for full models, not for the interventions that comprised them. These limitations made it impossible for decision makers to draw conclusions about which model worked best, for which families, in which context, why, and how.

Replication is a core tenet of open science and the economic model on scaling. With three or more randomized trials for some models, home visiting might seem to have more replication than other fields. Only nine evidence-based models have favorable effects in two or more studies; thus, most models meet criteria for being evidence-based based on only one sample (Mathematica Policy Research, 2019). Furthermore, results for models with replication studies are often inconsistent. It is hard to interpret such results since replication studies often used different methods and even the models themselves or their implementation might be different across studies in ways that are not reported (Michalopoulos et al., 2013). As in the original studies, analytic strategies of replications often elevated the risk for false positive results.

### How Open Science Principles Could Inform Home Visiting Evolution Going Forward

Traditional home visiting research often fails to define, measure, and test mechanisms of change. As a result, the building of knowledge is slowed, opportunities for shared learning are missed, and the field struggles to understand what truly drives impacts within and across evidence-based models. The field needs a clear statement of theory-driven hypotheses to promote specification of mechanisms of change which will directly improve scaling stronger programs (Duggan, 2021a; Supplee & Duggan, 2019). If the knowledge creation system for home visiting research incentivized pre-registration, study protocols, and open data, we believe we could begin to address challenges in scaling home visiting programs.

First, the field should prioritize specifying the specific interventions within models (these are often referred to as core components) before the start of the study because alterations in them during the study affect statistical inference. This could be done within pre-registration or publishing of study protocols. Home visiting models vary greatly; many are comprehensive, aiming to improve multiple outcomes through multiple interventions implemented over long periods of time. As a result, it is hard to determine how specific home visiting interventions influence outcomes, and hard to achieve replication. Some home visiting interventions are loosely structured with limited specificity and explicit direction. This, too, is important to document, as it has implications for replication and subsequent scale and spread.

Intervention descriptions have rarely been shared prior to formal research and testing. While premature release of untested interventions may lead to unwarranted uptake and use, sharing such information would encourage scientific and practice community input, leading to more effective approaches. In home visiting, proprietary ownership of manuals and materials complicates publication of intervention descriptions and discourages sharing and collective involvement in refinement and improvement. The field needs more replication of findings within and across home visiting models to strengthen impacts at scale. To support replications, the field needs to share full descriptions of interventions, as specified in reporting guidelines such as TIDieR (Hoffmann et al., 2014) and RoHVER (Till et al., 2015).

The Home Visiting Applied Research Collaborative (HARC) is addressing this barrier with core support from the MIECHV Program. HARC is a research and development platform in the USA that focuses on interventions within home visiting and uses innovative methods to learn what works best, for whom, in what contexts, why, and how (hvresearch.org). Its Precision Paradigm — a framework incorporating interventions, mediators, and moderators — is the touchstone for this work. HARC brings home visiting stakeholders together to define framework components using a common language, building on the ontology work of the Human Behaviour-Change Project (Michie et al., 2020). The Paradigm is a work in progress, with draft taxonomies constructed from the behavior change intervention literature and refined by applying consensus building techniques to the expert opinion of varied home visiting stakeholder groups. A recent study demonstrated the utility of this approach for defining five evidence-based models’ behavioral pathways and behavior change techniques to promote good birth outcomes (Duggan et al., 2021b). Current work builds on this initial study by assessing models — local program agreement on behavioral pathways and techniques, enrolled families’ views on specific behavior change techniques, and the adequacy of the home visiting literature in defining behavioral pathways and behavior change techniques in relation to reporting guidelines. The Paradigm’s shift from full models to interventions and its common framework, terminology, and definitions promote the field’s capacity for replication. In this way, the Precision Paradigm builds home visiting stakeholders’ capacity to embrace open science and the economic model on scaling.

Sharing of both study and routine program operations data is another beneficial feature of open science. It promotes accountability by providing a way to compare actual with planned implementation and creates opportunities for others to replicate and expand on original analyses. Barriers to data sharing within home visiting include lack of resources to prepare datasets and codebooks, investigator concerns that data might be analyzed incorrectly, an issue that is likely to be greater for proprietary interventions. Such barriers thwart the independent reproducibility of findings. As HARC shifts the focus from full home visiting models to generically defined interventions within them, data sharing barriers should diminish. In a current example, ten home visiting models in a Community of Practice are building their capacity for collaborative research using their existing management information system data (Sturmfels et al., 2021).

The practice of open science such as preregistration, study protocols, and open data would dramatically improve the available science to inform the scaling of home visiting. One example of the use of open science practices in home visiting is the Mother and Infant Home Visiting Program Evaluation (MIHOPE) Study, a large-scale, federally mandated multi-site clinical trial of four home visiting models implemented at scale (Michalopoulos et al., 2013). The study is particularly high-profile and politically sensitive, which made the use of open sciences practices even more valuable to build trust and transparency with the findings. First, the study published a design report prior to beginning any data collection or recruitment that was posted online, available for comment and a revised study protocol was posted online (Michalopoulos et al., 2013). The final study design was pre-registered at clinicaltrials.gov (Mother and Infant Home Visiting Program Evaluation - Full Text View - https://clinicaltrials.gov/ct2/show/NCT02069782). Second, all of the proposed measures and procedures were posted online to receive public comment (2013-00592.pdf (https://www.govinfo.gov/content/pkg/FR-2012-03-23/pdf/2012-6977.pdf)). Third, all the reports posted online include pre-specified confirmatory and exploratory outcomes and the results of all analyses, irrespective of whether they were null, negative, or positive (Michalopoulous et al., 2019). Finally, the study has made the data available for analysis (Warren, 2021). All of these practices together strengthened the transparency and trust in the findings and provided critical information on the true effects of programs at scale. The field still has substantial progress to make and open science can help.

## Discussion

The economic model on scaling provides the field an important framework to consider the breadth of science necessary to move the prevention field forward. More research is needed on all four parts of the economic model of scaling to ensure the promise of scaling evidence-based programs across prevention science can be delivered to communities. Within three of the five threats of the model, open science practices are central to ensuring the research generated has the best chance of maintaining trust in science as well as producing higher quality research.

Home visiting, as an example of a prevention program that has been scaled, is well-poised to take advantage of the open science movement. It is a widely disseminated preventive service strategy with committed stakeholders in the practice, research, and policy fields. There is a strong desire to improve home visiting impacts. Embracing open science, however, will require new ways of conducting research and engagement with the larger science and practice communities. The MIHOPE evaluation and work underway within HARC provide examples for the field to advance open science principles. Transparency, accountability, and sharing of protocols and data will all need to become standard practices if we are to accelerate the pace of knowledge accrual and leverage the collective expertise of our colleagues.

We believe it is imperative to move away from almost exclusive research on stand alone, multicomponent models and instead focus on (1) identifying specific intervention elements and mechanisms of change and (2) determining “what works best for whom in what contexts” through examination of intervention moderators (i.e., properties of the population and situation). Precision home visiting research encapsulates this approach and provides a roadmap for future research (Supplee & Duggan, 2019). With its emphasis on shared intervention design and testing, precision home visiting research is highly compatible with the goals of open science. Home visiting is fortunate that investment in the USA has made possible not only the expansion of evidence-based home visiting but also the building of critical research infrastructure, such as HARC, to advance the field. Transformative advances in home visiting are possible with research on commonly defined cross-model intervention elements and tailored approaches in a way that engages the field in all levels of the process. Open science can strengthen the rigor and utility of such research and advance effective scaling in the process. Early childhood home visiting provides a unique case study to better understand how open science could advance scaling programs, although learnings from the application of the economic model to this approach have broad applications to prevention science as a whole.

## Footnotes

1 One notable exception is the special symposium published in the *Journal of Economic Perspectives*, which included papers from both the development community (Banerjee et al., 2017; Muralidharan & Niehaus, 2017) and general lessons gleaned from the medication adherence literature for scaling (Al-Ubayldi et al., 2017).

2 Following this line of work (see, e.g., Al-Ubaydli et al. (2020b)), List and Suskind view three areas of import in the science creation market: (i) funding basic research (see List, 2011); (ii) providing the knowledge creation market with the optimal incentives for researchers to design, implement, and report scientific results; and (iii) developing a system whereby policymakers have the appropriate incentives to adopt effective policies, and once adopted develop strategies to implement those policies with rigorous evaluation methods to ensure continual improvement (see, e.g., Komro et al., 2016; Chambers et al., 2013). We focus on the second area in this paper but the other two are equally influential.

## Declarations

### Disclaimer

This information or content and conclusions are those of the author and should not be construed as the official position or policy of, nor should any endorsements be inferred by HRSA, HHS, or the US Government.
